# Highly Sensitive and Multiplexed In-Situ Protein Profiling with Cleavable Fluorescent Streptavidin

**DOI:** 10.3390/cells9040852

**Published:** 2020-04-01

**Authors:** Renjie Liao, Thai Pham, Diego Mastroeni, Paul D. Coleman, Joshua Labaer, Jia Guo

**Affiliations:** 1 Biodesign Institute & School of Molecular Sciences, Arizona State University, Tempe, AZ 85287, USA; renjie.liao@asu.edu (R.L.); thpham7@asu.edu (T.P.); joshua.labaer@asu.edu (J.L.); 2 ASU-Banner Neurodegenerative Disease Research Center, Biodesign Institute and School of Life Sciences, Arizona State University, Tempe, AZ 85287, USA; diego.mastroeni@asu.edu (D.M.); paul.coleman@asu.edu (P.D.C.); 3 L.J. Roberts Center for Alzheimer’s Research, Banner Sun Health Research Institute, Sun City, AZ 85351, USA

**Keywords:** proteomics, immunofluorescence, immunohistochemistry

## Abstract

The ability to perform highly sensitive and multiplexed in-situ protein analysis is crucial to advance our understanding of normal physiology and disease pathogenesis. To achieve this goal, we here develop an approach using cleavable biotin-conjugated antibodies and cleavable fluorescent streptavidin (CFS). In this approach, protein targets are first recognized by the cleavable biotin-labeled antibodies. Subsequently, CFS is applied to stain the protein targets. Though layer-by-layer signal amplification using cleavable biotin-conjugated orthogonal antibodies and CSF, the protein detection sensitivity can be enhanced at least 10-fold, compared with the current in-situ proteomics methods. After imaging, the fluorophore and the biotin unbound to streptavidin are removed by chemical cleavage. The leftover streptavidin is blocked by biotin. Upon reiterative analysis cycles, a large number of different proteins with a wide range of expression levels can be profiled in individual cells at the optical resolution. Applying this approach, we have demonstrated that multiple proteins are unambiguously detected in the same set of cells, regardless of the protein analysis order. We have also shown that this method can be successfully applied to quantify proteins in formalin-fixed paraffin-embedded (FFPE) tissues.

## 1. Introduction

Multiplexed molecular profiling in single cells in situ holds great promise to reveal cell-to-cell variations, cell-microenvironment interactions and tissue architecture at the single cell level, which are masked by population-based measurements [1,2]. Various methods [3,4,5,6,7,8,9,10] have been developed for multiplexed single-cell analysis. An increasing number of studies have been focused on proteins, for their central roles in biological processes. Immunofluorescence (IF) is a well-established single-cell in-situ protein analysis platform. However, on each specimen, only a couple of proteins can be profiled by IF, due to spectral overlap of commonly available organic fluorophores [11].

To enable multiplexed in-situ protein profiling, a number of methods [12,13,14,15,16,17,18,19,20] have been developed recently. In these methods, the detection tags are either conjugated to the primary antibodies or the secondary antibodies. Without signal amplification, the existing methods have limited detection sensitivity, which limits the analysis of low-expression proteins. Moreover, the low sensitivity is exacerbated in highly autofluorescent, formalin-fixed, paraffin-embedded (FFPE) tissues [13], which are the most common type of archived clinical tissue samples [21]. Additionally, due to their weak sensitivity, the current methods require long imaging exposure times, which results in limited sample throughput and long assay times.

Here, we report a highly sensitive and multiplexed in-situ protein analysis method. In this approach, protein targets are sensitively detected by cleavable biotin-conjugated antibodies and cleavable fluorescent streptavidin (CFS) using a layer-by-layer signal amplification method. Through reiterative cycles of protein labeling, signal amplification, fluorescence imaging, signal removal and streptavidin blocking, comprehensive protein profiling can be achieved in individual cells at the optical resolution. To demonstrate the feasibility of this approach, we designed and synthesized cleavable biotin-conjugated antibodies and CFS. We showed that the detection sensitivity of our approach is at least one order of magnitude higher than the current in-situ proteomics methods. We demonstrated that our approach enables accurate multiplexed protein analysis in single cells, without prior knowledge of the protein expression levels. We also showed proteins in FFPE tissues can be successfully profiled using our approach.

## 2. Materials and Methods

### 2.1. General Information

Chemicals and solvents were purchased from Sigma-Aldrich or TCI America, and used directly without further purification, unless otherwise noted. Bioreagents were purchased from Invitrogen, unless otherwise indicated.

### 2.2. Cell Culture

HeLa CCL-2 cells (ATCC) were maintained in Dulbelcco’s modified Eagle’s Medium (DMEM) supplemented with 10% fetal bovine serum, 100 U/mL penicillin and 100 g/mL streptomycin in a humidified atmosphere at 37 °C with 5% CO_2_. Cells were plated on chambered coverglass (0.2 mL medium/chamber) (Thermo Fisher Scientific) and allowed to reach 60% confluency in 1–2 days.

### 2.3. Cell Fixation and Permeabilization

Cultured HeLa CCL-2 cells were fixed with 4% formaldehyde (Polysciences) in 1X PBS (phosphate buffered saline) at 37 °C for 15 min, followed by washing with 1X PBS for 3 × 5 min. Cells were then permeabilized with PBT (0.1% Triton-X 100 in 1X PBS) for 10 min at room temperature, and subsequently washed three times with 1X PBS, each for 5 min.

### 2.4. Preparation of Cleavable Fluorescent Streptavidin

Cleavable Cy5 NHS ester was prepared according to the literature [14]. To 20 μL of streptavidin solution at a concentration of 1 mg/mL, 1 nmol of cleavable Cy5 NHS ester and 2 μL of NaHCO_3_ solution (1 M) were added. The mixture was incubated in the dark and at room temperature for 15 min. The labeled streptavidin was purified by p-6 biogel column.

### 2.5. Preparation of Biotin-SS-Ab

To 20 μL of primary antibody solution at a concentration of 1 mg/mL, 3 nmol of EZ link Sulfo-NHS-SS-Biotin (Thermo Fisher Scientific) and 2 μL of NaHCO_3_ solution (1 M) were added. The mixture was incubated in the dark and at room temperature for 15 min, and then the conjugation product was purified by p-6 biogel column.

### 2.6. Immunofluorescence with CFS

Fixed HeLa cells were incubated with antibody-blocking buffer (10% normal goat serum (*v/v*), 1% bovine serum albumin (*w/v*), 0.1 Triton-X 100 in 1X PBS) for 1 h at room temperature, and then washed three times with PBT, each for 5 min. To block the cell endogenous biotin, the cells were treated with 0.1 mg/mL streptavidin in 1X PBS for 15 min at room temperature, and washed three times with 1X PBS, each for 5 min. Subsequently, the cells were incubated with 0.5 mg/mL biotin in 1X PBS for 30 min at room temperature, and washed with 1X PBS three times, each for 5 min. After blocking, the cells were incubated with Biotin-SS-Ab in antibody-blocking buffer (concentration varied and was suggested by the manufacturers) for 45 min at room temperature, and washed with PBT three times, each for 10 min. Subsequently, the cells were incubated with 10 μg/mL cleavable fluorescent streptavidin in 1% BSA (bovine serum albumin) in PBT for 30 min, and washed three times with 1X PBS, each for 5 min. The cells were washed with GLOX buffer (0.4% glucose and 10 mM Tris HCl in 2 X SSC) for 1–2 min at room temperature, and then imaged in GLOX solution (0.37 mg mL^−1^ glucose oxidase and 1% catalase in GLOX buffer).

### 2.7. Signal Amplification

To amplify the staining signal, the cells were incubated with cleavable biotin-conjugated goat anti-chicken antibodies (Thermo Fisher Scientific) in 1% BSA in PBT at a concentration of 10 μg/mL for 30 min, and then washed three times with 1X PBS, each for 5 min. Afterwards, the cells were incubated with cleavable fluorescent streptavidin in 1% BSA in PBT at a concentration of 10 μg/mL, and again washed three times with 1X PBS, each for 5 min. Multiple amplification cycles can be repeated to obtain the desired signal intensity.

### 2.8. Fluorophore and Biotin Cleavage

Fluorophore and biotin cleavage was performed by incubating the specimen with tris(2-carboxyethyl)phosphine (TCEP, pH = 9.5, 100 mM in deionized water) for 30 min at 37 °C. Subsequently, the cells were washed three times with PBT and three times with 1X PBS, each for 5 min.

### 2.9. Streptavidin Blocking

After cleavage, the cells were incubated with 0.5 mg/mL biotin in 1X PBS for 30 min at room temperature, and then washed three times with 1X PBS, each for 5 min.

### 2.10. Quantification of the Fluorophore Cleavage Efficiency

Fixed and blocked HeLa CCL-2 cells were incubated with 10 μg/mL cleavable biotin-labeled rabbit anti-Ki67 (Thermo Fisher Scientific) for 45 min. Subsequently, the cells were stained by 10 μg/mL cleavable fluorescent streptavidin. Then, one, two, three and four rounds of amplification were applied to different sets of cells. In each round of amplification, the cells were first incubated with cleavable biotin-labeled goat-anti-chicken antibodies and then with cleavable fluorescent streptavidin. The cells were then incubated with TCEP (100 mM, pH = 9.5) for 30 min at 37 °C. Subsequently, the cells were washed three times with PBT and three times with 1X PBS, each for 5 min.

### 2.11. Quantification of the Biotin Cleavage Efficiency

Fixed and blocked HeLa CCL-2 cells were incubated with 10 μg/mL cleavable biotin-labeled rabbit anti-Ki67 (Thermo Fisher Scientific) for 45 min. Subsequently, cells were stained by 10 μg/mL cleavable fluorescent streptavidin. Following that, one, two, three and four rounds of amplification were applied to different sets of cells. In each round of amplification, the cells were first incubated with cleavable biotin-labeled goat-anti-chicken antibodies and then with cleavable fluorescent streptavidin. Biotin and fluorophores were cleaved by TCEP (100 mM, pH = 9.5). The cells were then incubated with cleavable fluorescent streptavidin.

### 2.12. Quantification of the Streptavidin Blocking Efficiency

Fixed and blocked HeLa CCL-2 cells were incubated with 10 μg/mL cleavable biotin-labeled rabbit anti-Ki67 (Thermo Fisher Scientific) for 45 min. Subsequently, cells were stained by 10 μg/mL cleavable fluorescent streptavidin. Following that, one, two, three and four rounds of amplification were applied to different sets of cells. In each round of amplification, the cells were first incubated with cleavable biotin-labeled goat-anti-chicken antibodies and then with cleavable fluorescent streptavidin. Biotin and fluorophores were cleaved by TCEP (100 mM, pH = 9.5). The cells were blocked with 0.5 mg/mL biotin. The cells were incubated with cleavable biotin-labeled goat anti-chicken and then cleavable fluorescent streptavidin.

### 2.13. Multiplexed Protein Analysis in HeLa Cells

Fixed and blocked HeLa CCL-2 cells were incubated with 10 μg/mL cleavable biotin-labeled primary antibodies. Subsequently, cells were stained by 10 μg/mL cleavable fluorescent streptavidin. One to three amplification cycles were applied. In each amplification cycle, cells were first incubated with cleavable biotin-labeled goat anti-chicken antibodies (Thermo Fisher Scientific) and then cleavable fluorescent streptavidin. After imaging, cells were incubated with TCEP (100 mM, pH = 9.5) to cleave the fluorophores and biotin. Cells were then blocked with 50 mM iodoacetamide, 0.1 mg/mL streptavidin and 0.5 mg/mL biotin, followed by the next immunofluorescence cycle. Rabbit anti-c-erbB-2 (Thermo Fisher Scientific), rabbit anti-Ki67 (Thermo Fisher Scientific), and rabbit anti-Histone H4 (mono methyl K20) (Abcam) were used as primary antibodies.

### 2.14. Conventional Immunofluorescence

Fixed and blocked HeLa CCL-2 cells were incubated with 10 μg/mL Cy5-labeled or unconjugated primary antibodies. Subsequently, cells were stained by 10 μg/mL Cy5-labeled goat anti-rabbit secondary antibodies (Thermo Fisher Scientific). Rabbit anti-c-erbB-2, rabbit anti-Ki67, and rabbit anti-Histone H4 (mono methyl K20) were used as primary antibodies. Cy5-labeled rabbit anti-Ki67 was prepared according to the literature [14].

### 2.15. Deparaffinization and Antigen Retrieval of FFPE Tissues

A brain FFPE tissue slide was deparaffinized in xylene three times, for 10 min each. Then the slide was immersed in 100% ethanol for 2 min, 95% ethanol for 1 min, 70% ethanol for 1 min, 50% ethanol for 1 min, 30% ethanol for 1 min. The slide was rinsed with deionized water. Afterwards, a combination of ‘heat induced antigen retrieval’ (HIAR) and ‘enzymatic antigen retrieval’ was used. HIAR was done using a pressure cooker (Cuisinart). The slide was immersed in antigen retrieval buffer (10 mM sodium citrate, 0.05% Tween 20, pH = 6.0), and water-bathed in pressure cooker for 20 min with the ‘High pressure’ setting. Subsequently, the slide was rinsed three times with 1X PBS, each for 5 min. The slides were treated with pepsin digest-all 3 (Life Technologies) for 10 min, and then washed three times with 1X PBS, each for 5 min.

### 2.16. Protein Staining in FFPE Tissues

To block the endogenous biotin, the slide was treated with 0.1 mg/mL streptavidin in 1X PBS for 15 min at room temperature, and washed three times with 1X PBS, each for 5 min. Subsequently, the slides were incubated with 0.5 mg/mL biotin in 1XPBS for 30 min at room temperature, and washed three times with 1X PBS, each for 5 min. The slide was incubated with 10 μg/mL cleavable biotin-labeled rabbit anti-H3K4me3 (Cells Signaling) in antibody blocking buffer for 45 min, and washed three times with PBT, each for 10 min. The slide was stained by 10 μg/mL cleavable fluorescent streptavidin for 30 min, and then washed three times with 1X PBS, each for 5 min. Two cycles of amplification were applied. In each round of amplification, the cells were first incubated with cleavable biotin-labeled goat-anti-chicken antibodies and then with cleavable fluorescent streptavidin. After imaging, the slide was incubated with TCEP (100 mM, pH = 9.5) for 30 min at 37 °C and washed three times with PBT and three times with 1X PBS, each for 5 min. Streptavidin was blocked with 0.5 mg/mL Biotin. The tissue was re-stained with 10 μg/mL cleavable biotin-labeled goat anti-chicken and then 10 μg/mL cleavable fluorescent streptavidin.

### 2.17. Imaging and Data Analysis

Stained cells and brain FFPE tissue were imaged under a Nikon Ti-E epifluorescence microscope equipped with a 20× objective. Images were captured using a CoolSNAP HQ2 camera and Chroma filter 49009. Image data was analyzed with NIS-Elements Imaging software.

## 3. Results

### 3.1. Platform Design

**Figure 1:**
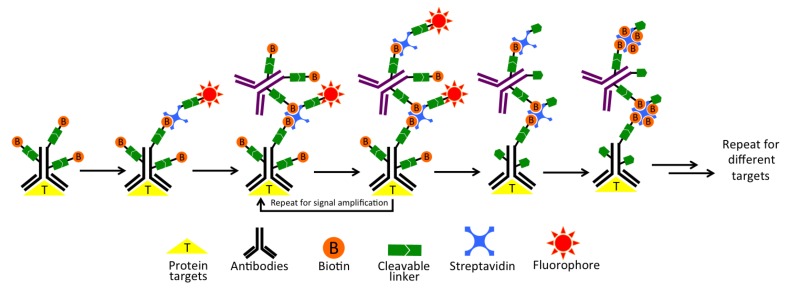
Highly sensitive and multiplexed in-situ protein profiling with cleavable fluorescent streptavidin (CFS). In each cycle, the protein of interest is first targeted by cleavable biotin-labeled primary antibodies, and then stained with CFS. Though layer-by-layer signal amplification using cleavable biotin-conjugated orthogonal antibodies and CFS, highly sensitive protein detection is achieved. After imaging, the fluorophore and the biotin unbound to streptavidin are chemically cleaved and subsequently streptavidin is blocked by biotin. Through reiterative cycles of target staining, signal amplification, fluorescence imaging, chemical cleavage and streptavidin blocking, comprehensive protein profiling can be achieved.

As shown in Figure 1, each staining cycle of our multiplexed protein profiling technology is composed of five major steps. First, proteins of interest are targeted by cleavable biotin-labeled primary antibodies and cleavable fluorescent streptavidin (CFS). Second, the specimen is incubated with a cleavable biotin-labeled orthogonal antibody or protein, which does not bind to any specific targets in the specimen or the primary antibodies. Then, CFS can be applied again to amplify the signal. This second step can be repeated several times to achieve the desired signal intensities through layer-by-layer signal amplification. Third, the specimen is imaged to generate quantitative single-cell protein expression profiles. Fourth, the fluorophore and the biotin unbound to streptavidin are efficiently removed by chemical cleavage. Finally, the leftover streptavidin is blocked with biotin. Through reiterative cycles of staining, amplification, imaging, cleavage and streptavidin blocking, a large number of different proteins with a wide range of expression levels can be characterized in single cells in situ.

### 3.2. Design and Synthesis of Cleavable Biotin-Conjugated Antibodies and CFS

To demonstrate the feasibility of this approach, we conjugated biotin to antibodies through a disulfide-bond-based cleavable linker and Cy5 to streptavidin through an azide-based cleavable linker, according to a previously described method [14]. In this way, both biotin and Cy5 can be simultaneously removed by the reducing reagent tris(2-carboxyethyl)-phosphine (TCEP).

### 3.3. Significantly Enhanced Detection Sensitivity

**Figure 2:**
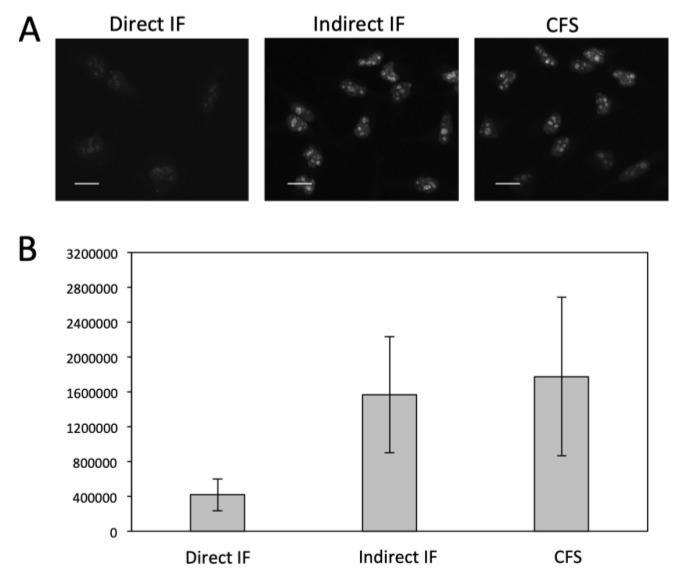
(**A**) Fluorescent images of protein Ki67 stained with direct IF, indirect IF and cleavable fluorescent streptavidin (CFS). Scale bars, 20 μm. (**B**) Comparison of the averaged signal integration in single cells (*n* = 30) for the three methods. Error bars, standard deviation.

**Figure 3:**
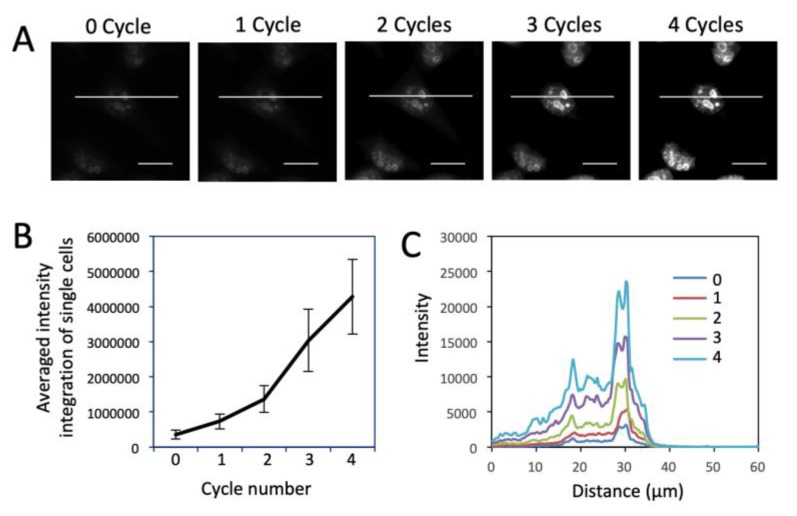
(**A**) Fluorescent images of protein Ki67 stained with 0 to 4 amplification cycles in HeLa cells. Scale bars, 20 μm. (**B**) Averaged signal integration in single cells (*n* = 30) in amplification cycles 0 to 4. Error bars, standard deviation. (**C**) Fluorescence intensity profiles corresponding to the indicated line positions in amplification cycles 0 to 4.

We then evaluated the detection sensitivity of our approach by comparing it with direct and indirect immunofluorescence (IF). Protein Ki67 in HeLa cells was stained with these three methods with the same concentration of primary antibodies (Figure 2). The staining patterns obtained by the three methods closely resemble each other. Compared with direct and indirect immunofluorescence, the CFS method does not lose the staining resolution (Figure 2A). Without any signal amplification steps, the CFS method is ~4.5 times more sensitive than direct immunofluorescence (*p* = 6.6e-5) and is comparable to indirect immunofluorescence (*p* = 0.36) (Figure 2B). With four rounds of signal amplification, the original staining intensities were further increased by more than 10 times (*p* = 3.8e-12) (Figure 3), while the staining background remained almost the same (Figure 3C). These results demonstrate that our approach has at least one order of magnitude higher detection sensitivity compared with the existing in-situ proteomics methods. The staining patterns obtained by direct IF, indirect IF and our approach closely resemble each other (Figure 2A and Figure 3A), suggesting that our signal amplification method does not lose the staining resolution.

### 3.4. Efficient Fluorophore and Biotin Cleavage and Streptavidin Blocking

**Figure 4:**
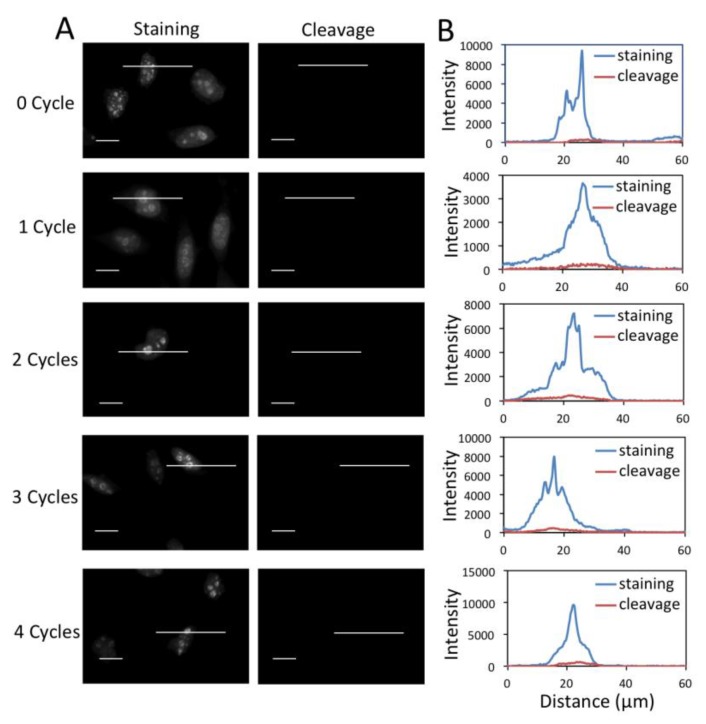
(**A**) Fluorescent images of protein Ki67 stained with 0 to 4 amplification cycles in HeLa cells and those after cleavage. The exposure time in amplification cycles 0 to 4 is 1 s, 500 ms, 250 ms, 125 ms, 62 ms, respectively. Scale bars, 20 μm. (**B**) Fluorescence intensity profiles corresponding to the indicated line positions in amplification cycles 0 to 4.

**Figure 5:**
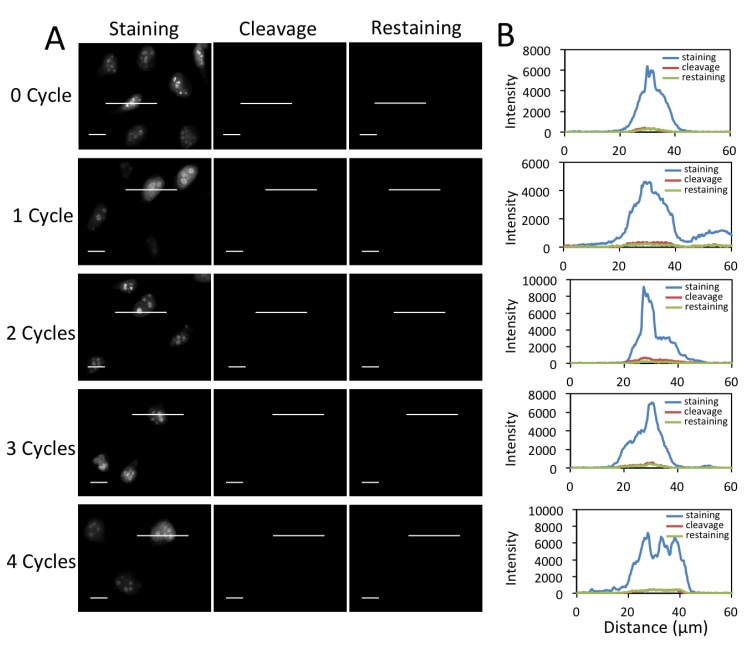
(**A**) Fluorescent images of protein Ki67 stained with 0 to 4 amplification cycles in HeLa cells, after cleavage and re-stained with CFS. The exposure time in amplification cycles 0 to 4 is 1 s, 500 ms, 250 ms, 125 ms, 62 ms, respectively. Scale bars, 20 μm. (**B**) Fluorescence intensity profiles corresponding to the indicated line positions in amplification cycles 0 to 4.

**Figure 6:**
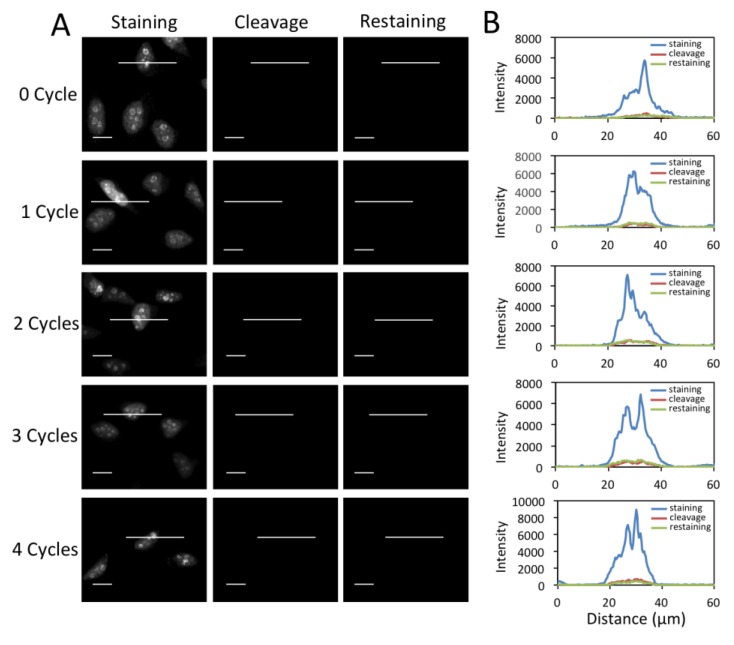
(**A**) Fluorescent images of protein Ki67 stained with 0 to 4 amplification cycles in HeLa cells and those after cleavage. Following streptavidin blocking, the cells were re-stained with cleavable biotin-labeled orthogonal antibodies and CFS. The exposure time in amplification cycles 0 to 4 is 1 s, 500 ms, 250 ms, 125 ms, 62 ms, respectively. Scale bars, 20 μm. (**B**) Fluorescence intensity profiles corresponding to the indicated line positions in amplification cycles 0 to 4.

To enable multiplexed protein analysis by reiterative analysis cycles, three major requirements exist. (1) Fluorescence signals need to be efficiently erased by chemical cleavage. (2) The biotin not bound to streptavidin has to be efficiently removed to avoid false positive signals in the next staining cycle. (3) As TCEP can not effectively cleave the biotin bound to streptavidin (data not shown), the free binding sites on the leftover streptavidin need to be efficiently blocked before the next staining cycle. To assess whether these three requirements are met by the CFS approach, we stained protein Ki67 with 0 to 4 amplification cycles in different sets of HeLa cells, and first quantified the cleavage efficiency (Figure 4). After TCEP incubation, ~95% of signal was removed regardless of the number of amplification rounds. To test whether the biotin unbound to streptavidin can be removed by TCEP, we stained protein Ki67 with 0 to 4 amplification cycles in different sets of HeLa cells (Figure 5). After TCEP cleavage, the cells were incubated the CFS again. No further fluorescence signal enhancement was introduced, suggesting that the free biotin is efficiently removed during the cleavage step. To evaluate the streptavidin blocking efficiency, we stained protein Ki67 with 0 to 4 amplification cycles in different sets of HeLa cells (Figure 6). Subsequently, the cells were incubated with TCEP and then with biotin to block streptavidin. Another round of signal amplification was applied and no further fluorescence signal enhancement was detected. These results indicate that streptavidin is efficiently blocked by biotin.

### 3.5. Multiplexed In-Situ Protein Profiling

**Figure 7:**
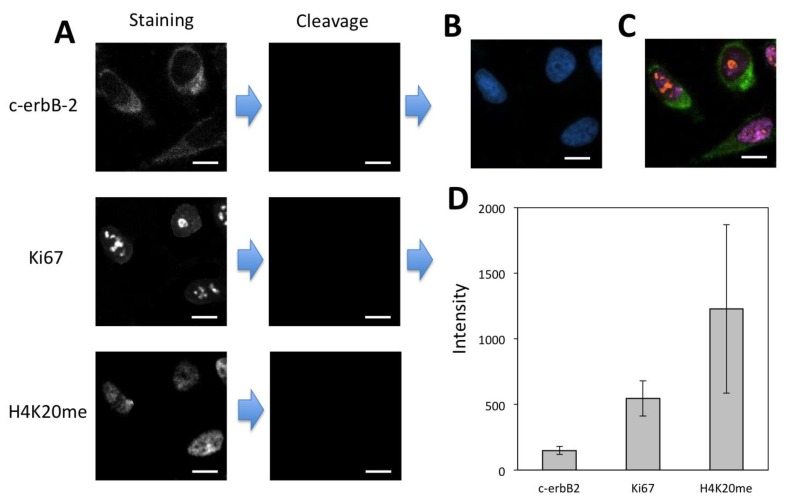
(**A**) Protein c-erbB-2, Ki67 and H4K20me were detected with CFS through reiterative staining cycles in the same set of HeLa cells. (**B**) Nuclei were stained with DAPI. (**C**) Digital overlay of the three staining images in (**A**). (**D**) Staining intensity (*n* = 40) for the three proteins. Error bars, standard deviation. Scale bars, 20 μm.

To demonstrate the feasibility of applying the CFS method for multiplexed protein analysis, we labeled protein c-erbB-2, Ki67 and H4K20me through reiterative staining cycles in the same set of HeLa cells (Figure 7). The staining signals generated in the previous cycles do not reappear in the following cycles, confirming that the fluorophore and free biotin are efficiently cleaved and streptavidin is efficiently blocked. We also stained these three proteins by conventional immunofluorescence (Figure S1). The staining results obtained by our approach and conventional immunofluorescence closely resemble each other. These results suggest that the CFS method enables the multiplexed protein profiling in single cells in situ.

**Figure 8:**
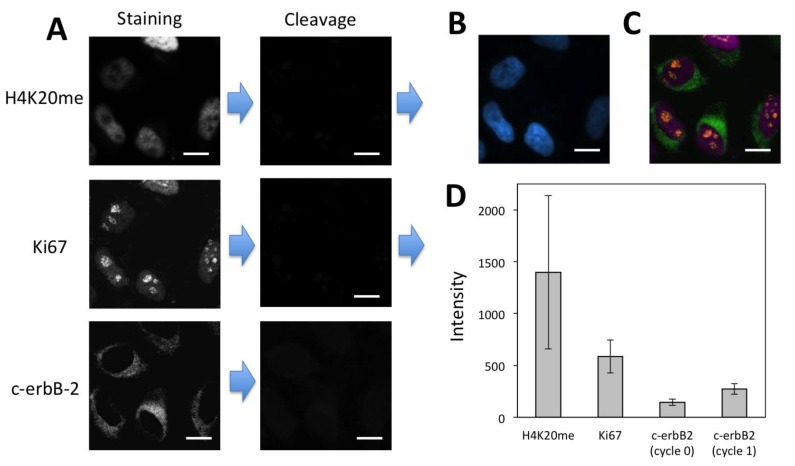
(**A**) H4K20me and Ki67 were detected with CFS through reiterative staining cycles without signal amplification. Afterwards, protein c-erbB-2 was detected by signal amplification with CFS in the same set of HeLa cells. (**B**) Nuclei were stained with DAPI. (**C**) Digital overlay of the three staining images in (**A**). (**D**) Staining intensity (*n* = 40) for the three proteins. Error bars, standard deviation. Scale bars, 20 μm.

Existing reiterative protein profiling methods [12,13,14,15,16,17,18,19] require knowledge of the relative protein expression levels in advance. With that prior knowledge, proteins are quantified in the order of their increasing expression levels, to minimize the interference from the leftover signals generated in the previous cycles. However, due to the limited amount of the biological and clinical samples, to obtain prior knowledge of protein expression levels is sometimes not possible. In addition, the relative protein expression levels in different cell types in the same specimen can be different, which makes it difficult to develop a desired protein analysis order for all the cell types. Moreover, the process to generate such prior knowledge can be time-consuming and expensive. Our CSF method addresses all of these issues by eliminating the requirement of knowing protein expression levels in advance. In our approach, the protein staining signal in each analysis cycle can be amplified with a certain number of amplification cycles until the satisfied staining intensities are achieved. In this way, following the analysis of high-expression proteins in the previous cycles, the low-expression proteins can be accurately quantified by more amplification cycles. To demonstrate the feasibility of this concept, we profiled protein H4K20me, Ki67 and c-erbB-2 in the same set of HeLa cells in the order of decreasing expression levels (Figure 8A–C). As a result of efficient fluorophore and biotin cleavage and also efficient streptavidin blocking, protein Ki67 was successfully detected following the analysis of high-expression H4K20me. However, due to the extremely low expression level of c-erbB-2 and the accumulated leftover signals produced in the previous two cycles, it was difficult to detect protein c-erbB-2 without signal amplification (Figure S2). After one cycle of signal amplification, the staining signal of c-erbB-2 was significantly enhanced. The significant stochastic protein expression heterogeneity resulted in the relatively large error bars in Figure 7D and Figure 8D [14]. With the improved signal-to-background ratio, the low-expression c-erbB-2 was unambiguously detected following the analysis of two high-expression proteins. These results indicate that the CFS method does not require the prior knowledge of protein expression levels and enables accurate protein analysis regardless of the protein analysis order.

### 3.6. In-Situ Protein Profiling in Human FFPE Tissues

**Figure 9:**
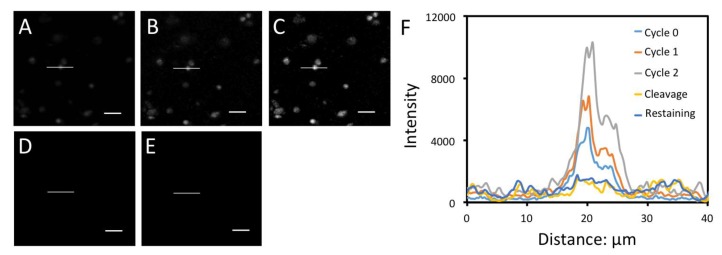
Protein H3K4me3 in human FFPE brain tissue was stained with CFS in amplification cycles (**A**) 0, (**B**) 1 and (**C**) 2. (**D**) Afterwards, the stained tissue was incubated with TCEP. (**E**) Following chemical cleavage and streptavidin blocking, the tissue was incubated with cleavable biotin-conjugated antibodies and CFS again. (**F**) Fluorescence intensity profiles corresponding to the indicated line positions in (**A**) to (**E**). Scale bars, 25 μm.

Archived tissues are important biological samples to study normal physiology and disease pathogenesis. Formalin-fixed, paraffin-embedded (FFPE) tissue is the most common form of archived tissue in clinics and pathology labs [21]. FFPE tissues often display high autofluorescence [13] and partially degraded proteins [22], which makes them difficult to profile by fluorescence imaging methods with low detection sensitivity. To demonstrate the feasibility of applying the CFS approach to analyze FFPE tissues, we stained H3K4me3 in FFPE human brain tissue (Figure 9). With two rounds of signal amplification, the signal-to-background ratio was significantly improved. After cleavage, the fluorescence signal was efficiently removed. Another round of signal amplification cycle after cleavage and streptavidin blocking did not further increase the staining intensities. These results confirm that the flurophore and the free biotin can be efficiently removed by TCEP and streptavidin can be efficiently blocked by biotin. These results also imply that the CFS approach can be successfully applied to quantify the proteins in FFPE tissues.

## 4. Discussion

In summary, we have designed and synthesized CFS and demonstrated that this multiplexed in-situ protein analysis approach enhances the detection sensitivity of the existing in-situ proteomics approaches by at least ten times. This improved sensitivity enables our approach to precisely quantify the low-expression proteins, which are not detectable by other current in-situ proteomics methods. In this way, our approach also improves the dynamic range of protein detection by one order of magnitude. We have also shown that multiple proteins can be accurately detected in the same specimen using our approach, regardless of the analysis order of proteins with varied expression levels. With its dramatically-improved sensitivity, our approach enables the quantitative analysis of low-expression proteins, especially in the highly autofluorescent FFPE tissue samples.

Similarly to other in-situ proteomics assays, our approach applies the signal intensities generated by the target-bound antibodies to infer the relative abundances of the proteins. As a result, it can be difficult to compare the results obtained using different antibodies with varied binding affinities and specificities. To precisely quantify the amount of proteins in the sample, mass spectrometry can be applied first, to determine the absolute copy number of the proteins in standard cells. Then, these standard cells can be analyzed together with the sample of interest using our approach. By calibrating the generated results with the standard cells, we can calculate the exact copy number of the protein target in the sample.

The multiplexing capacity of this approach depends on two factors: the number of reiterative analysis cycles and the number of proteins quantified in each cycle. We have shown previously that protein antigenicity is preserved after incubation with TCEP for at least 24 h [14], which suggests that more than 40 cycles can be carried out on the same specimen. In each cycle, varied protein targets can be first recognized by primary antibodies labeled with distinct cleavable haptens, such as biotin, fluorescein, TAMRA, and digoxigenin (DIG). Subsequently, streptavidin, anti-fluorescein, anti-TAMRA, and anti-DIG antibodies labeled with different fluorophores can be applied to stain the protein targets and amplify the signals. In this way, at least four proteins can be quantified simultaneously in each cycle. Thus, we anticipate this method has the potential to analyze over 100 protein targets in the same specimen.

In addition to protein profiling, this cleavable layer-by-layer signal amplification approach developed here can also be applied for highly sensitive in-situ DNA [6], RNA [23,24] and metabolic analysis [25]. By combining these applications, integrated in-situ genomics, proteomics and metabolomics analysis can be achieved in the same specimen at the optical resolution. This highly sensitive and multiplexed molecular imaging platform will have wide applications in systems biology and biomedical research.

## Supplementary Materials

The following are available online at https://www.mdpi.com/2073-4409/9/4/852/s1, Figure S1: Fluorescent images obtained by conventional immunofluorescence; Figure S2: Fluorescent image of protein c-erbB-2 stained in the third analysis cycle without signal amplification.
