# Supplementary material for: Highly Sensitive and Multiplexed In-Situ Protein Profiling with Cleavable Fluorescent Streptavidin
Source: Cells. 2020 Apr 1;9(4):852. doi: 10.3390/cells9040852 (PMC7226835; doi:10.3390/cells9040852)
Supplement: Supplementary file 1 [file cells-09-00852-s001.pdf]

Supplementary Materials

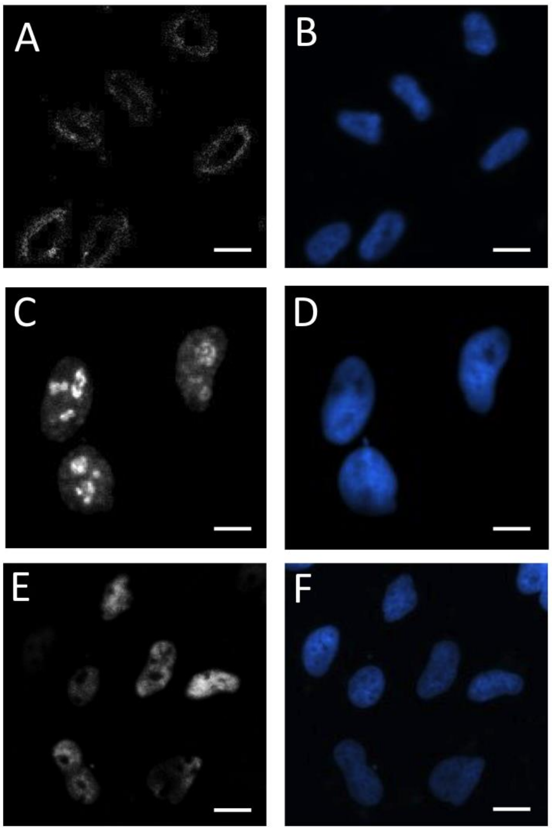

Figure S1. Fluorescent images obtained by conventional immunofluorescence. (**A**) Fluorescent image of protein c-erbB-2 in HeLa cells by conventional immunofluorescence and (**B**) the corresponding DAPI staining of nuclei. (**C**) Fluorescent image of protein Ki67 in HeLa cells by conventional immunofluorescence and (**D**) the corresponding DAPI staining of nuclei. (**E**) Fluorescent image of protein H4K20me in HeLa cells by conventional immunofluorescence and (**F**) the corresponding DAPI staining of nuclei. Scale bars, 20 μm.

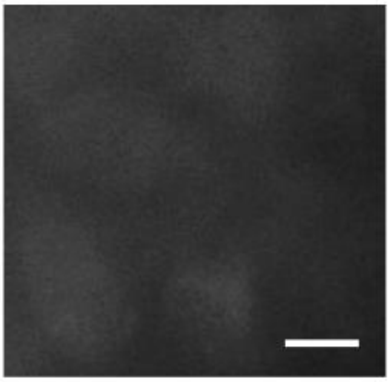

Figure S2. Fluorescent image of protein c-erbB-2 stained in the third analysis cycle without signal amplification. Scale bar, 20 μm.
